# Data mining of cancer vaccine trials: a bird's-eye view

**DOI:** 10.1186/1745-7580-4-7

**Published:** 2008-12-12

**Authors:** Xiaohong Cao, Karen B Maloney, Vladimir Brusic

**Affiliations:** 1 Babson College, Wellesley, MA, USA; 2 Cancer Vaccine Center, Dana-Farber Cancer Institute, Boston, MA, USA

## Abstract

**Background:**

A wealth of information on clinical trials has been provided by publicly accessible online registries. Information technology and data exchange standards enable rapid extraction, summarization, and visualization of information and derived knowledge from these data sets. Clinical trials data was extracted in the XML format from the National Library of Medicine ClinicalTrials.gov site. This data includes categories such as 'Summary of Purpose', 'Trial Sponsor', 'Phase of the Trial', 'Recruiting Status', and 'Location'. We focused on 645 clinical trials related to cancer vaccines. Additional facts on cancer types, including incidence and survival rates, were retrieved from the National Cancer Institute Surveillance data.

**Results:**

This application enables rapid extraction of information about institutions, diseases, clinical approaches, clinical trials dates, predominant cancer types in the trials, clinical opportunities and pharmaceutical market coverage. Presentation of results is facilitated by visualization tools that summarize the landscape of ongoing and completed cancer vaccine trials. Our summaries show the number of clinical vaccine trials per cancer type, over time, by phase, by lead sponsors, as well as trial activity relative to cancer type and survival data. We also have identified cancers that are neglected in the cancer vaccine field: bladder, liver, pancreatic, stomach, esophageal, and all of the low-incidence cancers.

**Conclusion:**

We have developed a data mining approach that enables rapid extraction of complex data from the major clinical trial repository. Summarization and visualization of these data represents a cost-effective means of making informed decisions about future cancer vaccine clinical trials.

## Background

The World Health Organization's Global Burden of Disease statistics identified cancer as the second largest global cause of death, after cardiovascular disease [1]. Cancer is the fastest growing segment of the disease burden; global cancer deaths are projected to increase from 7.1 million in 2002 to 11.5 million in 2030 [2]. Advances in prevention, diagnostics and treatment of cancer have contributed to the improved prognosis for cancer patients: one third of cancers are preventable and another third are curable through early detection and effective therapy [3]. New cancer therapies are subject of vigorous research including the application of new high-throughput biomedical technologies that generate large amounts of data. The related information explosion mandates the use of biomedical bioinformatics.

Researchers and clinicians need rapid access to multiple types of information, including molecular, clinical, and literature databases, and clinical trials registries, as well as suitable data analysis tools. The National Center for Biotechnology Information (NCBI) hosts resources for retrieval and analysis of bio-molecular data. These are accessible through the NCBI's web site [4]. Clinical trials data are available through clinical trials registries. Since 1971, regulatory efforts on clinical trials registration have resulted in a significant level of compliance by both sponsors and conductors of clinical trials [5]. ClinicalTrials.gov [6] has emerged as the largest registry in the world [7]. Currently it contains information on 50,000 clinical trials including 16,000 cancer-related entries. The US National Cancer Institute (NCI) provides access to a clinical trials registry within the Physician Data Query (PDQ) database [8]. The PDQ contains (Dec 2007) 21,000 abstracts of cancer-related clinical trials and regularly exchanges data with the ClinicalTrials.gov registry. Data sharing and direct access to resources (researchers, computers, software, data, research participants, and other) are considered critical for the advancement of cancer research and the improvement of health care. The initiatives such as Cancer Research Network [9] and Cancer Biomedical Informatics Grid [10] provide the framework for integration of various data types and tools for cancer research. Standardized data formats (*e.g*. demographics, health plan eligibility, tumor registry, inpatient and ambulatory utilization, medication dispensing, laboratory tests, imaging procedures, others) [9] facilitate access to and sharing of data and automated analysis.

Knowledge discovery from databases, also known as data mining, is an emerging field that applies techniques from databases, statistics and artificial intelligence to extract high-level information (knowledge) from a large volume of low-level data. Examples of high-level information derived from low-level data include forms that are more compact (*e.g*., short reports), more abstract (*e.g*., descriptive models of the process that generated data), or more useful (*e.g*., predictive models for estimating values of the future cases) than existing low-level data [11]. Mining clinical trials data usually refers to using statistical and modeling tools for analysis and design of clinical trials. If appropriate clinical trials data (*e.g*. aims, goals, regimens and conditions, end points, sample sizes, and others) are stored in the registry, data mining can help design better, more efficient trials that require smaller patient cohorts. Standardized data formats, such as XML markup language [12], bring text files into machine-readable form, thus enhancing automated analysis. Both NCI PDQ and ClinicalTrials.gov provide clinical trial registry data in the XML format.

We have developed a data mining approach for rapid summarization and visualization of information from clinical trial registries. This method has been applied to the analysis of cancer vaccine trials and provides convenient means of extraction and presentation of key data about cancer vaccine trials. The significant progress in cancer biology and cancer immunology has not yet been fully translated into successful clinical vaccine applications [13]. Though advances in cancer vaccine development have been reported [*e.g*. [14,15]], a wide variety of factors are involved in tumor immune escape, making design and production of effective therapeutic vaccines difficult. Increasing knowledge of possible limiting factors include, among others, dysfunction of the immune system, immunosuppressive effects of tumor microenvironment, production of suppressor T cells, defective antigen processing and presentation, and immunotherapy resistance of established tumors. To utilize the accumulated data and knowledge and translate these into improved clinical trials, integration of basic and clinical immunology and improved data processing capabilities is required. Our data mining approach provides better understanding of the cancer vaccine clinical trials landscape, and enables rapid analysis of the hotspots of cancer vaccine activity, as well as the identification of neglected cancers. This report describes the utility of basic data mining techniques of summarization, tabulation, and visualization applied to the clinical trials repository data.

## Results

### Cancer vaccine trials data mining questions

A key characteristic of our application is its versatility; questions can be formulated and re-formulated rapidly. A complex, composite question also can be defined directly from the interface using several mouse clicks. This application has been designed to dynamically generate graphs, summarizing data across relevant clinical trials.

Our application supports formulating basic questions and queries. A representative, yet not exhaustive list of basic questions and queries is shown in Table 1. Answers to questions such as "How has the cancer vaccine field evolved in the last ten years?" and "How many cancer vaccine trials have been conducted; how many of them are currently open in the United States?" offer a historical view of the vaccine trials field. Similarly, answers to questions like "What cancer types are currently researched in clinical trials?" and "What phase are these trials?" offer an up-to-date view of the cancer vaccine field. In addition, this application helps answer more specific questions such as "How many breast cancer vaccine trials were conducted by Dana-Farber Cancer Institute's Cancer Vaccine Center and what types of vaccines were used for those trials?"

**Table 1:** Sample questions that can be answered by this system.

**Examples of cancer vaccine trials landscape questions:**
1. How many vaccine trials have started each year since 1995?
2. How many of these trials are currently open?
3. Who are the lead sponsors for these trials?
4. What are the main vaccine platforms used by these sponsors in the trials?
5. Do they focus on certain cancer types or on specific vaccine platform?
6. What are the cancer types that have been interrogated using vaccine strategy?
7. What are the cancer types that are currently interrogated?
8. What phase are these trials?
9. Is there any linkage between cancer prevalence and the numbers of vaccine trials for that particular cancer?
10. Are there any cancers neglected within the vaccine field?

Representative questions specific to the cancer vaccine field are shown. Because of the large number of searchable terms and their combinations

The versatility of this system enables the analysis of various dimensions of the clinical trials landscape, including clinical trials by timeline, type of cancer, lead institution, patient population, and/or specific vaccine technology.

### Trials over time

**Figure 1:**
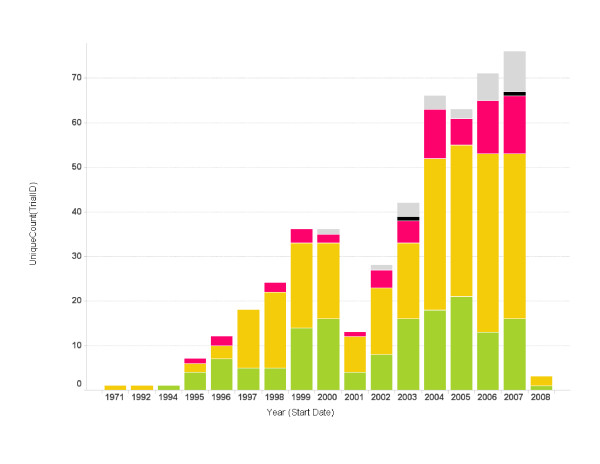
**Cancer vaccine trials started per year**. The number of trials started each year is shown. Bars represent the total number of trials started for a particular year. The color code on each bar represents the phase of the trials (green: phase 1; yellow: phase 2; red: phase 3; grey: unspecified).

Figure 1 summarizes the number of clinical cancer vaccine trials conducted in the US each year during the last 30 years. The first autologous tumor cell vaccine was used in a lung cancer trial as early as 1971 at the University of Medicine and Dentistry in New Jersey. Clinical trials using vaccine technology gradually re-emerged in the early 1990s, and the field grew rapidly until 2000, with more than 30 vaccine trials starting in that year. Since then new cancer vaccine trials have shown a steady increase reaching more than 60 new vaccine trials each year. Most of the earlier trials were phase I and II. The number of phase III clinical vaccine trials has increased steadily during the past three years.

### Types of cancers most often targeted by vaccine strategy

Clinical vaccine trials by cancer type and trial phase are shown in Table 2. The top five cancers targeted by vaccine therapy in clinical trials are: melanoma, cervical, prostate, breast, and leukemia. Melanoma has attracted by far the most attention among cancers; nearly a quarter of all clinical vaccine trials target melanoma. Cervical cancer is second, with 10% of total vaccine trials. In addition, each of prostate, leukemia, breast, lung, lymphoma, and kidney cancers has been studied in more than twenty clinical vaccine trials. Phase III clinical vaccine trials of cervical cancer vaccines represent the largest group making 47% of the total number trials.

**Table 2:** Vaccine trial counts by cancer types and trial phase.

**Cancer Type**	**Phase 1**	**Phase 2**	**Phase 3**	**Phase 4**	**Unspecified**	**Total**
Melanoma	43	107	9		7	166
Cervix	6	23	35	1	5	70
Breast	37	27	1		4	69
Prostate	20	37	4		3	64
Lung	25	28	5		2	60
Ovary	24	11	1		3	39
Non-Hodgkin lymphoma	7	20	7	1	2	37
Colon	11	22			3	36
Kidney	7	23	2			32
Rectum	9	18			3	30
Pancreas	8	17	3		1	29
CML	9	14			1	24
AML	9	10	1		3	23
Myeloma	5	12	2		2	21
Brain	11	8	1			20
Eye	4	14			2	20
Other oral cavity	10	5				15
Pharynx	10	4			1	15
Mouth	10	4				14
All sites	10	3			1	14
Liver	5	7			2	14
CLL	8	5				13
Soft tissue	7	5			1	13
Stomach	8	3				11
Esophagus	6	2				8
Hodgkin lymphoma	2	5			1	8
ALL	3	2			2	7
Bladder	5		1		1	7
Bone	3	2			1	6
Vulva	1	4				5
Testis	3	1				4
Vagina		4				4
Cranial nerves & other nervous system	1	1			1	3
Gallbladder & other billiary	2	1				3
Small intestine	1	1			1	3
Anus	1					1
Corpus		1				1
Other non-epithelial skin	1				0	1
Penis	1					1
Tongue		1				1
Total	189	348	72	2	34	645

The number of vaccine trials per cancer type and trial phase are illustrated. The columns are sorted by total trial number per cancer.

### Institutions and corporations most active in developing cancer vaccines

**Figure 2:**
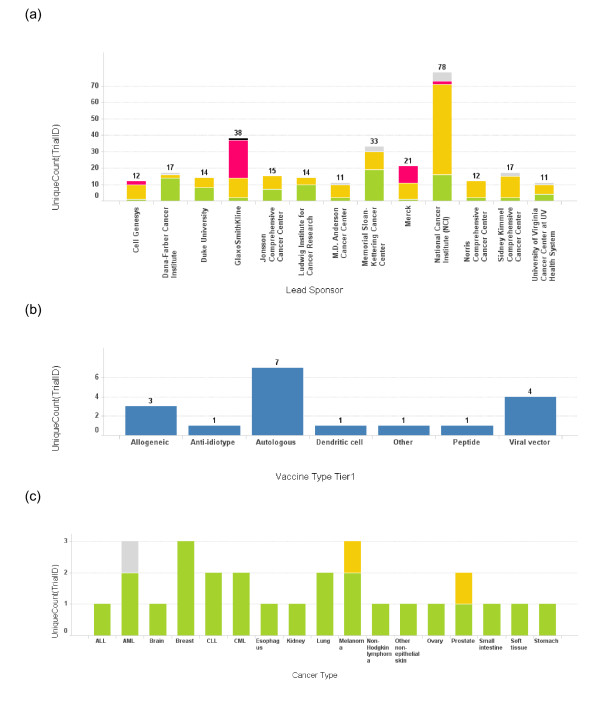
**Number of trials conducted by each lead sponsor**. (a) The number of cancer vaccine trials conducted by each lead sponsor is plotted as a bar graph by the name of the sponsor. Only the sponsors who have conducted more than 10 cancer vaccine trials are depicted here. The height of the bar and the number on top of each bar represent the total number of trials for a sponsor. The bars are also color coded according to the phase of the trials. Green: phase 1; Yellow: phase 2; Red: phase 3; Grey: phase unspecified. (b) A bar graph depicts the vaccine platform profile for Dana-Farber Cancer Institute. The bar heights represent the number of the trials conducted using each vaccine platform. (c) A bar graph depicts cancer types targeted in cancer vaccine trials at Dana-Farber Cancer Institute. The bar heights represent the number of the trials and the bars are color coded using the same color scheme as 3(a).

The breakdown of institutions that are major lead sponsors of cancer vaccine trials, the total number of trials, and trial phases, are shown in Figure 2a. The cancer centers that are included in this list are well known for provision of comprehensive cancer care and treatments. The results show that, to date, phase III cancer vaccine trials have been sponsored almost exclusively by pharmaceutical corporations. The exceptions are two phase III trials sponsored by the NCI. The graphs representing various activities and details can be generated by the data mining application. Examples of generated graphs are shown in Figure 2: a) activities by the leading players (institutions that have sponsored more than ten clinical trials each within the vaccine field, b) number of vaccine trials without trial phase information, by technology platform for Dana-Farber Cancer Institute (DFCI), and c) types of cancer with trial phases sponsored by DFCI. Similar graphs depicting the number of cancer vaccine trials can be generated for the following queries: type of cancer, phase of clinical trial, sponsor, and vaccine technology. Other queries can be easily formulated.

### Trials by disease prevalence

**Figure 3:**
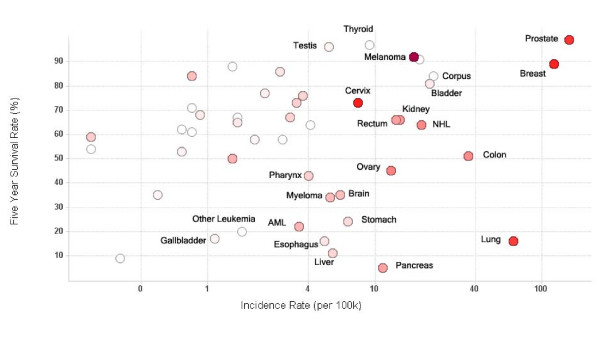
**Vaccine trials activity relative to cancer prevalence and survival**. Scatter plot of five year survival rate against cancer incidence rate for major cancers. Each circle represents an individual cancer type. The incidence rate (cases per 100,000 population) on the x-axis is log scaled for visual clarity. The red color of the circles represents the number of trials that have been conducted for that cancer type – the higher the number, the darker the circle. Grey circles represent cancer types which have not been studied using clinical vaccine trials. Selected cancer types are labeled.

In the previous section, we have identified the cancer types most often targeted by vaccine strategies, representing actively-supported areas of cancer vaccine trials. We also wanted to know vaccine trial activities for other cancer types. Figure 3 displays the scatter plot of the 5 year survival rate against the incidence rate for various cancer types. This figure shows that most of clinical vaccine trials target cancers with high incidence rates, such as breast, prostate and lung cancers. In addition, most clinical vaccine trials target cancers associated with high rates of 5-year survival. This figure clearly shows the "neglected cancers" within the cancer vaccine landscape, including bladder, liver, pancreatic, stomach, esophageal, and all of the low-incidence cancers.

### Technology platforms used by vaccine strategies

**Figure 4:**
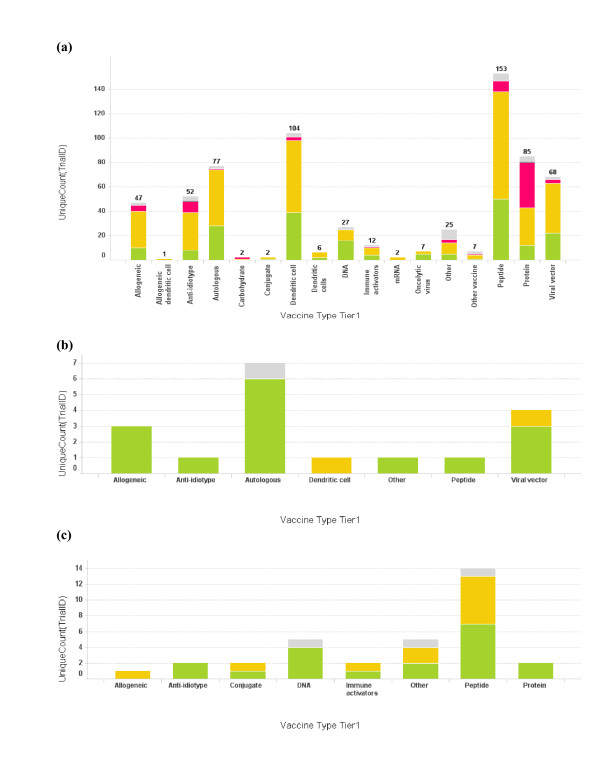
**Profile of vaccine technologies used in vaccine trials**. (a) Bar graph of total number of cancer vaccine trials conducted for each vaccine platform. The bar height and the number on top of the graph represent trial count, though single vaccine trials may at times incorporate multiple vaccine platforms; (b) Vaccine platforms for Dana-Farber Cancer Institute vaccine trials; (c) Vaccine platforms for Memorial Sloan-Kettering Cancer Center. The color coding is the same as used in Fig 1. When the vaccine technology is not known for the trial, it is labeled as "Empty".

The analysis of technology platforms used in the vaccine trials is shown in Figure 4a. It is not surprising to see that the majority of the trials use antigens to directly stimulate host immune response against cancer cells. These antigen vaccines can be full length proteins or truncated (peptide) forms. Cellular vaccine trials are second in number to antigen-based vaccine trials; together they make some 80% of the total. Currently most of the cell vaccines are made from autologous cells harvested from patients. However, allogeneic cell vaccines are becoming more popular in the last three years (data not shown). We also observed that individual institutions preferentially pursue a small number of cancer vaccine strategies. For illustration, DFCI pursues mainly cellular or viral technology strategies in developing their vaccine platforms (Figure 4b), while Sloan Kettering Memorial Cancer Center focuses mainly on antigen-based vaccines (Figure 4c).

## Conclusion

A large number of records are available in clinical trial repositories but the analysis is limited to simple queries. This data mining application for summarization and visualization of clinical trials data was developed and applied to the analysis of cancer vaccine clinical trials landscape. It enables complex queries of cancer vaccine trials data through data mining. Data mining revealed hidden patterns, trends and biases in the data. The application enables formulating queries and asking specific questions. It revealed the cancer vaccine trials development over time. The most common cancers targeted by vaccine therapy in clinical trials are: melanoma, cervical, prostate, breast, and leukemia. Neglected cancers include bladder, liver, pancreatic, stomach, esophageal, and all of the low-incidence cancers. Our approach provides a bird's-eye view of the cancer vaccine landscape, and enables rapid analysis of the hotspots of cancer vaccine activity, as well as the identification of neglected cancers.

## Discussion

The landscape of clinical vaccine trials has multiple dimensions. These include an historical timeline of cancer vaccine trials, organizations sponsoring vaccine trials, principal technologies, and types of cancers, all of which we analyzed in this study. Many of the fields in the XML files were not included in this study, for example, "minimum age", "maximum age", "inclusion criteria", "exclusion criteria", and others. The selection of fields was defined by the scope of this study: rapid extraction of clinical trials statistics and identification of major activities in the field.

For studies that focus on other questions, such as patient groups, different sets of questions would be asked and different dimensions would be included for selections of relevant fields from the XML files.

The decision by the US Food and Drug Administration (FDA) to publicize information on clinical trials was made to enable public access to data that address questions about the safety of certain drugs [5]. In addition, public access to this data helps counter publication bias, in which positive results are published more easily and faster than negative results [5]. Though a significant quantity of data and information that describe cancer vaccine clinical trials is available, the existing means of converting these data into knowledge are less advanced.

Our data mining process consisted of data preparation and the application of summarization [see [11]] and visualization [see [16]] tools upon extracted specific knowledge from cancer vaccine clinical trials data. By accessing comprehensive clinical trials information using appropriate software tools, several mouse clicks provide access to knowledge that would otherwise require hiring of specialists or consultants. This knowledge is useful for various professionals, including research directors and planners in companies, hospital administrators, researchers and scientists, physicians conducting clinical trials, policy makers, grant officers at funding bodies, and government regulators. By combining public databases of clinical trials, data formatting by XML, and computational analysis and visualization, we have shown that specific knowledge can be extracted, summarized, and presented to the user.

We have implemented a highly flexible system to visualize and mine clinical trial information in the cancer vaccine field. We obtained all data about cancer vaccine trials from ClinicalTrials.gov database. In accordance with the 1997 FDA Modernization Act [17], information from clinical trials on serious or life-threatening diseases must be registered at ClinicalTrials.gov within 21 days of the trial starting date. The ClinicalTrials.gov has become the world's largest clinical trial database and it contains information from clinical trials performed world-wide. Although some cancer vaccine trials may not necessarily be included, ClinicalTrials.gov repository is comprehensive and representative of the world-wide landscape of clinical trials in the cancer vaccine field. Some fields of interest in this study were not included in the original XML files; for example, fields related to specific vaccine technology. We inspected the records, identified vaccine technology, and manually annotated this field using external data sources. In addition, clinical trials records in the back-end database were enriched with fields containing cancer statistics data. Manual annotation of these data has limitations, principally because subsequent studies also need manual annotation. This shortcoming can be addressed either by automation of annotation, or by inclusion of these fields in the clinical trials records. We envision that the power of this system can only increase with the improvement of the content of public databases.

## Methods

### Data

Of 16,000 cancer trials in ClinicalTrials.gov, some 900 are cancer vaccine or cancer immunotherapy trials. The information provided in the trial descriptions includes "summary of purpose", "trial sponsor", "phase of the trial", "recruiting status", and "location", and other, more specific information. Clinical trials related to cancer vaccines are defined by keywords "cancer" and "vaccine" present in the title or abstract of the trial record. Prior to January 2008, some 642 trials fit this definition. Input primary data consisted of downloaded XML files containing information on cancer vaccine trials.

Additional information on cancer types was retrieved from the Surveillance Epidemiology and End Results (SEER) Cancer Statistics Review (CSR) provided by the National Cancer Institute (NCI). Incidence and survival rates associated with various cancer types were obtained from the SEER/CSR report 2003 [18].

### System description

**Figure 5:**
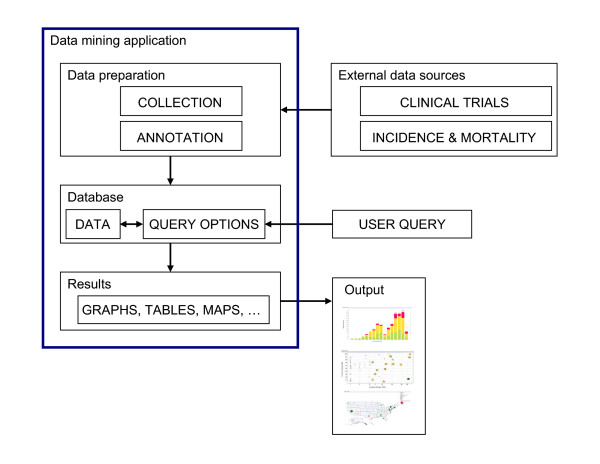
**Overview of the system and workflow**. A flowchart of the work flow to establish the system is shown here. Data relevant to each cancer vaccine trial and cancer prevalence data were obtained from public sources and were then further annotated to form an XML database. Desired data were then retrieved and reformatted before loading into Spotfire DXP for visualization. Various graphs were generated with each iterative cycle of querying and re-querying until a final collection of informative graphs was generated.

Our data mining system consists of a back-end XML database, a front-end visualization interface, and the analysis component. The analysis workflow is shown in Figure 5. First, XML files for relevant cancer vaccine trials were downloaded from the ClinicalTrials.gov website and incidence and survival facts were downloaded from the National Cancer Institute (NCI) website. We have defined a series of questions to address using this system. Fields of interest contain information, such as 'Cancer Type', 'Phase of the Trial', and 'Recruiting Status'; these fields were extracted from the primary XML files. Additional fields of interest, such as 'Technology Platform', 'Adjuvant Usage', and 'Therapy type', that provide information in form suitable for database querying, were added manually and associated to each clinical trial record in our back-end database. These data were not available as separate fields in the ClinicalTrials.gov records, but could be derived from the descriptions and mapped. Clinical trial records contain additional fields such as 'Minimum Age', 'Maximum Age', 'Inclusion Criteria', 'Exclusion Criteria', among others. That information was outside the scope of this study and was not included in the back-end database of our system. Nevertheless, these fields can easily be included in the data mining system if needed. The technology platform information is not available as a separate field in ClinicalTrials.gov but can be extracted from the content of other fields and interpreted using data from NCI Drug Dictionary [19]. The list of applicable technologies is shown in Table 3. Cancer statistics data are not available at all in the ClinicalTrials.gov and they were created from external data [18] [see Additional file 1]. The clinical trial data was merged with cancer statistical facts using "Cancer Type" as the link. Spotfire has the capability of importing additional data from an external file or database and we used this capability. Several graphical views were generated to answer specific questions.

**Table 3:** Major cancer vaccine technologies.

**Vaccine antigen status**	**Vaccine technology**
Defined antigen	Dendritic cell (peptide)
	Dendritic cell (protein)
	Dendritic cell (specific RNA)
	Dendritic cell (virus)
	Viral vector (antigen)
	Viral vector (other)
	Naked DNA
	Protein
	Peptides
	Anti-idiotype
	Antigen-specific adjuvant
	Other

Undefined antigen	Dendritic cells (tumor cell pulsed)
	Dendritic cells (lysate pulsed)
	Dendritic cells (allo-lysate pulsed)
	Allogeneic Dendritic cells (lysate pulsed)
	Dendritic cells (antigen)
	Dendritic cells (non-specific DNA)
	Dendritic cells (non-specific RNA)
	Dendritic cells (idiotype)
	Oncolytic virus
	Autologous (tumor lysate)
	Allogeneic (tumor lysate)
	Autologous (tumor cell)
	Allogeneic (tumor cell)
	Autologous (T cell)
	Autologous (lymphocytes)
	Autologous (PBMC)
	Immunomodulators
	Immune activators
	Other non-cancer vaccines
	Other

More details about technological platforms for cancer vaccines can be found in [20].

### Spotfire DXP Platform Description

We have chosen Spotfire-DXP (version 2.0, spotfire.tibco.com) software to construct the environment for our data mining application. Spotfire-DXP is proprietary software which can be licensed through TIBCO Software Inc. . The Spotfire-DXP is a dynamic tool suitable for summarization and visualization of tabular data. Although other similar tools, such as SAS, Insightful, and others offer more sophisticated statistical power, they are less suitable for summarization, visualization, and tabulation tasks. Spotfire is easy to learn and use because of graphical user interface that allows graphing and tabulation by using drag-and-drop actions.

The software environment consists of four main areas, 'Menu Bar', 'Main Graphing Area', 'Query Filter Panel', and 'Details on Demand'. The top 'Menu Bar' consists of file and data manipulation tools, in addition to icons for generating new graphs. The 'Main Graphing Area' is screen space where user displays results in either graphs or tables. The right 'Query Filter Panel' is automatically populated with column heads when the data table is brought in. The 'Details on Demand' window will only show up when a user wants to investigate a subset of records in more details.

**Figure 6:**
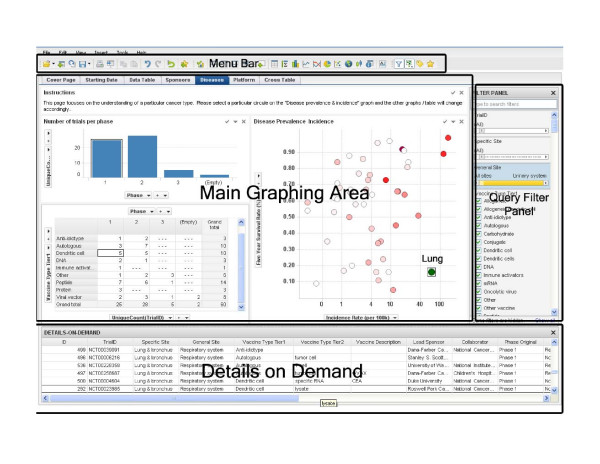
**Data mining framework**. A screenshot of the interface to our data mining system. The interface has four major sections: menu bar (top), query filter panel (right), database details on demand (bottom), and the main graphing area (central).

The interface with representative examples is illustrated in Figure 6. When the program is started with a selected data table, the top 'Menu Bar' and the 'Query Filter Panel' appear, along with the default cover page, and a scatter plot is automatically generated in the 'Main Graphing Area'. To change the axis of the graph, the user can simply drag the appropriate field from the filter panel onto the x or y axis. To change the type of graph plotted, the user can click on the "x" symbol at the top right corner of the "Main Graphing Area" to close the default scatter plot and click on another graph icon from the top "Menu Bar" to generate another graph. Axes can then be changed as described previously. Tables can also be generated using a similar drag-and-drop action. The properties of a particular graph can be changed through the property window by adjusting relevant parameters. Parameters that users can select include color, shape, size, and other graphical properties for displaying high dimension data. Once the graph is generated, the user can plot more detailed graphs by selecting a subset of data within 'Query Filter Panel'. The graph will change dynamically upon data filtering and the corresponding records will be displayed in the 'Details on Demand' window. The user can generate several graphs on a single page, as shown in Figure 6. These graphs are linked; records selected in one graph will also be highlighted in all other graphs. Formulated queries are stored as "pages" shown in the tabs under the toolbar ('Cover Page', 'Data Table', 'Lead Sponsors – Players', and others). The user can plot new graphs on a separate page by clicking on the 'New Page' icon on the top 'Menu Bar' and generate graphs as previously described.

### Data mining

Tabular data are constructed from the backend database. Our application uses standard Spotfire-DXP toolbox for extraction and manipulation of data. Fields that we used in this study included 'Cancer Type', 'Incidence Rate', '5-Year Survival (%)', 'Trial ID', 'Brief Summary', 'Lead Sponsor', 'Collaborator', 'Phase', 'Trial Status', 'Vaccine Platform', and 'Trial Start' data.

To illustrate the data mining process, we have used an example in which we answered Question 1 (Table 1) by generating a bar graph shown in Figure 1. The process started by selection of the 'Bar Graph' on the 'Menu Bar'. This generated the default bar graph in the 'Main Graphing Area'. Then, we selected the 'Start Date' field from the 'Query Filter Panel' and dragged it to the x-axis. Similarly, we dragged the 'TrialID' from the 'Query Filter Panel' to the y-axis. We clicked on the y-axis to apply the 'UniqueCount' function to the y-axis. Once this was done, we changed the coloring scheme by clicking the 'Property' icon in the 'Menu Bar' and specified the colors for different phases (green for phase 1, yellow for phase 2 and red for phase 3.). These actions produced the graph on the 'Trials Started per Year' page. Similarly, we constructed other pages that query specific information about lead sponsors, diseases, vaccine technology platform, and geographic locations, as shown in Figure 6. The data sets and application programs are available upon request from the authors.

## Competing interests

The authors declare that they have no competing interests.

## Authors' contributions

XC proposed the original idea and the design of the project, programmed and implemented the system. KBM provided guidance and contributed to the design of the project. VB contributed to the design of the project, and provided direction and technical expertise, and annotated records. The three authors contributed equally to the writing.

## Supplementary Material

Additional file 1 **Table with statistical data: new cases and deaths per annum, and five year survival per cancer type (US data)**. Click here for file
