# Supplementary material for: Data mining of cancer vaccine trials: a bird's-eye view
Source: Immunome Res. 2008 Dec 12;4:7. doi: 10.1186/1745-7580-4-7 (PMC2639543; doi:10.1186/1745-7580-4-7)
Supplement: Additional file 1 — Table with statistical data: new cases and deaths per annum, and five year survival per cancer type (US data). [file 1745-7580-4-7-S1.pdf]

Estimated new cases, deaths, and 5 year survival per cancer type per annum (USA data)

		Estimated new cases (USA, annual)			Estimated deaths (USA, annual)			5yr survival %
		Both sexes	Male	Female	Both sexes	Male	Female	
All sites		1,437,180	745,180	692,000	565,650	294,120	271,530	
Oral cavity & pharynx		35,310	25,310	10,000	7,590	5,210	2,380	0.6
	Tongue	10,140	7,280	2,860	1,880	1,210	670	0.58
	Mouth	10,820	6,590	4,230	1,840	1,120	720	0.73
	Pharynx	12,410	10,060	2,350	2,200	1,620	580	0.43
	Other oral cavity	1,940	1,380	560	1,670	1,260	410	0.59
Digestive system		271,290	148,560	122,730	135,130	74,850	60,280	0.44
	Esophagus	16,470	12,970	3,500	14,280	11,250	3,030	0.16
	Stomach	21,500	13,190	8,310	10,880	6,450	4,430	0.24
	Small intestine	6,110	3,200	2,910	1,110	580	530	0.58
	Colon	108,070	53,760	54,310	49,960	24,260	25,700	0.51
	Rectum	40,740	23,490	17,250				0.66
	Anus anal canal & anorectum	5,070	2,020	3,050	680	250	430	0.67
	Liver & intrahepatic bile duct	21,370	15,190	6,180	18,410	12,570	5,840	0.11
	Gallbladder & other biliary	9,520	4,500	5,020	3,340	1,250	2,090	0.17
	Pancreas	37,680	18,770	18,910	34,290	17,500	16,790	0.05
	Other digestive organs	4,760	1,470	3,290	2,180	740	1,440	0.09
Respiratory system		232,270	127,880	104,390	166,280	94,210	72,070	0.19
	Larynx	12,250	9,680	2,570	3,670	2,910	760	0.64
	Lung & bronchus	215,020	114,690	100,330	161,840	90,810	71,030	0.16
	Other respiratory organs	5,000	3,510	1,490	770	490	280	0.61
Bones & joints		2,380	1,270	1,110	1,470	820	650	0.68
	Bones & joints	2,380	1,270	1,110	1,470	820	650	0.68
Soft tissue (including heart)		10,390	5,720	4,670	3,680	1,880	1,800	0.67
	Soft tissue (including heart)	10,390	5,720	4,670	3,680	1,880	1,800	0.67
Skin (excluding basal & squamous)		67,720	38,150	29,570	11,200	7,360	3,840	0.91
	Melanoma	62,480	34,950	27,530	8,420	5,400	3,020	0.92
	Other non-epithelial skin	5,240	3,200	2,040	2,780	1,960	820	0.88
Breast		184,450	1,990	182,460	40,930	450	40,480	0.89
	Breast	184,450	1,990	182,460	40,930	450	40,480	0.89
Genital system		274,150	195,660	78,490	57,820	29,330	28,490	0.73
	Uterine cervix	11,070		11,070	3,870		3,870	0.84
	Uterine corpus	40,100		40,100	7,470		7,470	0.45
	Ovary	21,650		21,650	15,520		15,520	0.77
	Vulva	3,460		3,460	870		870	0.53
	Vagina & other genital female	2,210		2,210	760		760	0.99
	Prostate	186,320	186,320		28,660	28,660		0.96
	Testis	8,090	8,090		380	380		0.71
	Penis & other genital male	1,250	1,250		290	290		0.68
Urinary system		125,490	85,870	39,620	27,810	18,430	9,380	0.74

	Urinary bladder	68,810	51,230	17,580	14,100	9,950	4,150		0.81
	Kidney & renal pelvis	54,390	33,130	21,260	13,010	8,100	4,910		0.66
	Ureter & other urinary organs	2,290	1,510	780	700	380	320		0.54
Eye & orbit		2,390	1,340	1,050	240	130	110	0.84	
	Eye	2,390	1,340	1,050	240	130	110		0.84
Brain & other nervous system		21,810	11,780	10,030	13,070	7,420	5,650	0.35	
	Brain	21,810	11,780	10,030	13,070	7,420	5,650		0.35
Endocrine system		39,510	10,030	29,480	2,430	1,110	1,320	0.94	
	Thyroid	37,340	8,930	28,410	1,590	680	910		0.97
	Other endocrine	2,170	1,100	1,070	840	430	410		0.62
Lymphoma		74,340	39,850	34,490	20,510	10,490	10,020	0.68	
	Hodgkin lymphoma	8,220	4,400	3,820	1,350	700	650		0.86
	Non-Hodgkin lymphoma	66,120	35,450	30,670	19,160	9,790	9,370		0.64
Myeloma		19,920	11,190	8,730	10,690	5,640	5,050	0.34	
	Myeloma	19,920	11,190	8,730	10,690	5,640	5,050		0.34
Leukemia		44,270	25,180	19,090	21,710	12,460	9,250	0.5	
	Acute lymphocytic leukemia	5,430	3,220	2,210	1,460	800	660		0.65
	Chronic lymphocytic leukemia	15,110	8,750	6,360	4,390	2,600	1,790		0.76
	Acute myeloid leukemia	13,290	7,200	6,090	8,820	5,100	3,720		0.22
	Chronic myeloid leukemia	4,830	2,800	2,030	450	200	250		0.5
	Other leukemia	5,610	3,210	2,400	6,590	3,760	2,830		0.2
Other & unspecified primary sites		31,490	15,400	16,090	45,090	24,330	20,760	0.16	
	Other primary sites	31,490	15,400	16,090	45,090	24,330	20,760		0.16

Reference:

[1] American Cancer Society. *Cancer Facts and Figures 2008,* Atlanta: American Cancer Society, 2008.

[2] Ries LAG, Melbert D, Krapcho M, Stinchcomb DG, Howlader N, Horner MJ, Mariotto A, Miller BA, Feuer EJ, Altekruse SF, Lewis DR, Clegg L, Eisner MP, Reichman M, Edwards BK (eds). SEER Cancer Statistics Review, 1975-2005, National Cancer Institute. Bethesda, MD, 2008. http://seer.cancer.gov/csr/1975_2005
